# Assessment of air handling unit to improve ventilation in congregate living settings: a multicenter cross-sectional study

**DOI:** 10.1017/ice.2026.10458

**Published:** 2026-07

**Authors:** Christina K. Chan, Charlie Tan, Heather Candon, Matthew Crittenden, Michael McRitchie, James Callahan, Jeff Powis, Jerome A. Leis

**Affiliations:** 1 Sunnybrook Health Sciences Centre, Toronto, Ontario, Canada; 2 Air and Water Precision Balancing Incorporated, Toronto, Ontario, Canada; 3 Toronto East Health Network Michael Garron Hospital, Toronto, Ontario, Canada; 4 Department of Medicine and Centre for Quality Improvement and Patient Safety, Temerty Faculty of Medicine, University of Toronto, Toronto, Ontario, Canada

## Abstract

**Background:**

Heating, ventilation, and air conditioning (HVAC) systems can modulate the risk of respiratory virus transmission. In congregate living settings (CLS), variability in the quality of this infrastructure presents an improvement opportunity.

**Methods:**

We performed a cross-sectional study of the air handling units (AHU) of HVAC systems across a network of CLS to identify the most common problems and specific opportunities to improve ventilation. A third-party expert performed systematic HVAC assessments, which were analyzed based on presence and function of key AHU components to determine their association with air changes per hour (ACH) using a multivariable linear regression model.

**Results:**

Across 15 participating CLS, the median ACH was 3.3 (IQR = 10.4) in common areas, 3.5 (IQR = 12.6) in resident rooms, and 6.6 (IQR = 9.7) in resident bathrooms. Closed outside air dampers and fans turned off was associated with decreased ACH in resident rooms (−9.5 ACH, 95% CI, −0.2 to −18.9) and resident washrooms (−7.0 ACH, 95% CI, −1.3 to −12.7). The absence of ducted air source was associated with decreased ACH in resident rooms (−12.2 ACH, 95% CI, −1.0 to −23.5).

**Conclusions:**

Across 15 CLS, we identified readily actionable targets for improvements to ventilation. Based on our findings, we propose assessment criteria to optimize the performance of existing AHU. Prospective studies evaluating the impact of a standardized approach to improving AHU on safety of CLS residents are needed.

## Introduction

Suboptimal ventilation in congregate living settings (CLS), including nursing homes (NH) and independent living facilities (ILF), has been associated with spread of respiratory viruses and other adverse health effects among older and vulnerable resident populations.^1–4^

Regulatory bodies and expert organizations have issued guidance on the importance of evaluating and improving indoor ventilation as a fundamental infection prevention measure against respiratory viruses.^5,6^ Despite this, significant variability remains across CLS, particularly in presence of older and aging infrastructure of Heating Ventilation Air Conditioning (HVAC) systems.^7,8^ We recently systematically assessed ventilation and other physical characteristics across a large network of CLS in Toronto, Canada, and found that each additional air change per hour (ACH) was associated with an approximately 10% lower risk of transmission of SARS-CoV-2.^2^

CLS may face challenges in identifying actionable improvements to existing air handling units (AHU) to optimize ventilation and improve the safety of these environments. Due to lack of evidence linking specific AHU deficiencies with ACH, many improvements may instead rely on expert opinion.^5,6^ We performed the following cross-sectional study of the HVAC systems across our CLS network to identify the most common problems affecting AHUs that may represent actionable opportunities to improve ventilation.

## Methods

### Study setting and design

CLS in Toronto, Canada have been supported by hospital-based Infection Prevention and Control (IPAC) programs, known as IPAC Hubs, since October 2020.^9^ Among 15 CLS (8 NH and 7 ILF) supported by two IPAC Hubs covering north and east Toronto, physical measurements of representative rooms were undertaken from February to April, 2023, as previously described.^2^ These HVAC assessments were categorized based on room type including shared resident rooms, shared resident washrooms and common areas (e.g., dining rooms, activity rooms).^2^ In the current cross-sectional study, one room conformation by room type, specifically common area, resident room and resident washroom, was included per facility to identify the presence and function of specific AHU components and whether these were associated with reduced ventilation. Research ethics review was not required because the study met institutional criteria at both IPAC hubs for exemption as quality improvement research.

### AHU components and definitions

**Figure 1.**
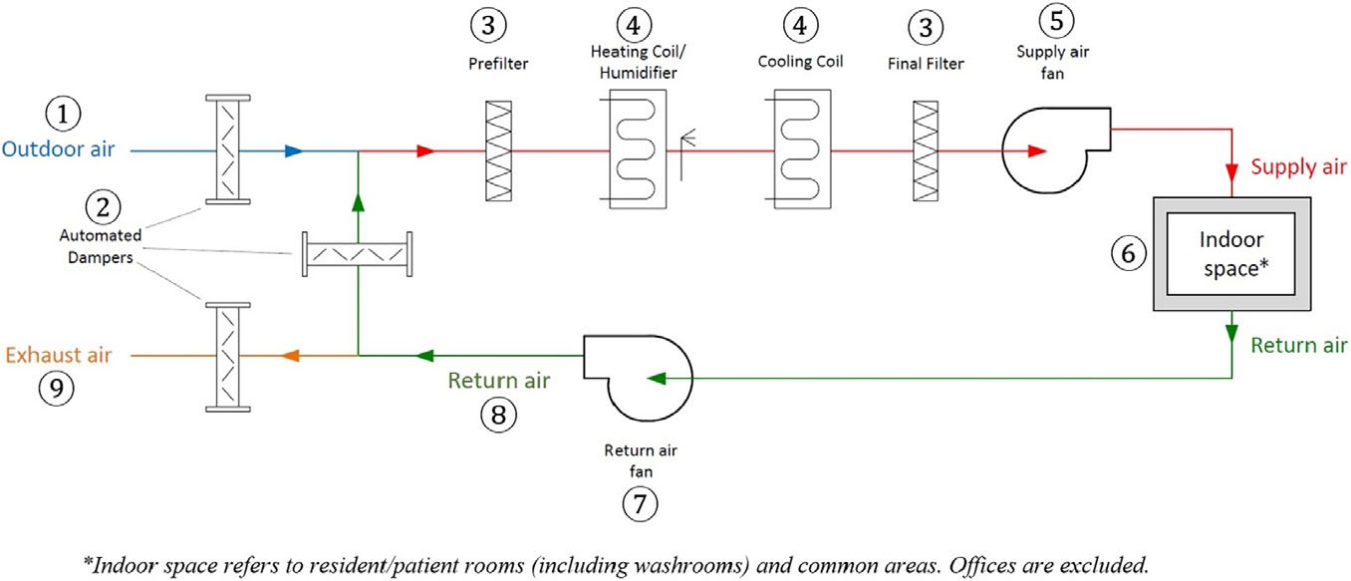
Figure 1 long description. Simplified schematic of an air handling unit (mixed air type).

A simplified schematic of an AHU is shown in Figure 1. For study purposes, the key AHU components were defined as follows. Outdoor air referred to air outside a building or taken from the external atmosphere and has not previously circulated through the AHU. Dampers were defined as elements inserted into an air-distribution system that permit modification of air resistance, thereby changing the airflow rate or shutting off the airflow. Prefilter was defined as the first stage of air filtration designed to remove large airborne particles. A heating coil referred to a coil that uses a heat transfer fluid, condensing refrigerant or direct electrical resistance elements to provide heating to heat fluids. A humidifier referred to a device adding moisture to the air. A cooling coil referred to an arrangement of pipes or tubes that can be used either with refrigerant or secondary coolant to provide cooling or cooling with dehumidification. Final filter was a filter positioned in the last filtering position in an AHU to collect dust that has passed through. Supply air was defined as the air delivered by mechanical or natural ventilation to a space, composed of any combination of outdoor air and recirculated air. Air ducts were defined as any tube or conduit used for conveying air to rooms. Return air was the air removed from a room to be recirculated or exhausted whereas exhaust air was defined as air that must be removed from a space due to contaminants, regardless of pressurization.^10^

### Study outcome and data collection

The primary outcome was the number of ACH by room type. ACH was measured using two methods: direct air volume readings at ceiling terminals with a Balometer capture hood (Shortridge Instruments ADM 870C) and velocity readings at grille-type terminals using a digital vane anemometer, with air volume conversion using correction factors (K-Factors).^2^ ACH values were extracted from HVAC reports and compared against established national reference standards: 6 ACH for common areas, 4 ACH for resident rooms, and 9 ACH for resident washrooms.^11^

To determine predictors of ACH, assessment of AHU components was reviewed across participating CLS. The HVAC system was categorized as having a central or non-central AHU, based on whether the AHU served multiple spaces from a central location or whether individual AHUs were installed closer to or within the room they served, including local heating or cooling units.^11^ Hybrid systems, which combine features of both central and non-central AHUs, were also identified where applicable. Data for each room type were extracted from HVAC reports for presence and function of key AHU components. These included 1) Outdoor air flow, evaluated by presence of ducted sources in resident rooms and connection to the AHU in common areas, 2) Dampers present including outdoor air dampers, return air dampers and exhaust air dampers, and whether these were open and maintained proper air balance and minimum outdoor air supply, 3) Filters present with adequate Minimum Efficiency Reporting Value (MERV) rating in all AHU, 4) Heating and cooling coils being clean and functioning properly in all AHU, 5) Supply air fan being operational in both resident rooms and common areas, 6) Return air fan being operational in both resident rooms and common areas; 7) Return air flow being present in both resident rooms and common areas, and 8) Exhaust air flow being present in common areas and resident bathrooms, controlled via independent or tied switches. To determine AHU function, equal weight was attributed to each of these 8 components, with multiple AHU deficiencies defined as absence or dysfunction of at least two or more of these components.

### Statistical analysis

Descriptive statistics included frequency, proportion, mean, standard deviation (SD), and range (minimum to maximum value). Boxplots were used to visually compare HVAC assessment across different CLS rooms. Multivariable linear regression analysis was performed to assess the association between ACH and AHU components. Given the importance of all variables, the model included type of air supply, damper closure or fan shutdown, inadequate filter, suboptimal non-continuous supply airflow, required maintenance, and absence of a ducted air source in resident rooms. A *P*- value of <.05 was considered significant. Statistical analysis was performed using STATA SE 18.0 (StataCorp, College Station, TX, USA).

## Results

Among the 15 CLS assessed, the median ACH was 3.3 (IQR = 10.4) in common areas, 3.5 (IQR = 12.6) in resident rooms and 6.6 (IQR = 9.7) in resident washrooms. Of these, 26.7% (4/15) of common areas, 46.7% (7/15) of resident rooms, 40.0% and (6/15) bathrooms met the existing standard.^11^

There was a central AHU in 4 CLS (26.7%), while a non-central or hybrid system was present in 2 (13.3%) and 9 (60.0%) CLS, respectively. Suboptimal non-continuous airflow, resulting from fan settings on “Auto” mode, was observed in 6 CLS (40.0%). With regard to assessment of specific AHU components, 4 CLS (26.7%) required replacement of filter media in AHU serving both resident rooms and common areas, 5 (33.3%) required servicing of AHU in any type of rooms and 4 (26.7%) had closed outside air dampers that may have restricted airflow in these areas and 5 (33.3%) lacked air ducts to resident rooms. Among resident washroom exhaust fans, 5 CLS (33.3%) had dedicated on/off switches, 3 (20.0%) were tied to the light and 1 (6.7%) operated with continuous airflow.

**Figure 2.**
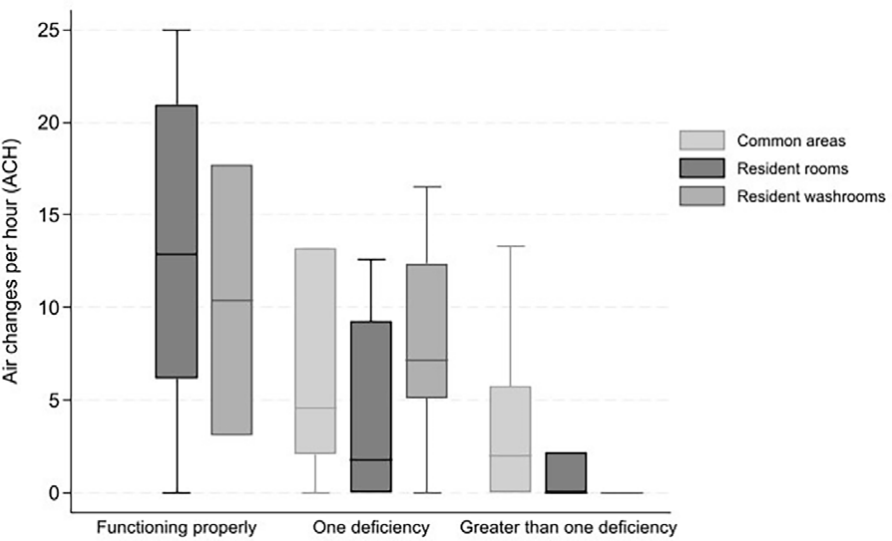
Median air changes per hour and interquartile range across different room types, and categorized based on the number of deficiencies in air handling unit.

Figure 2 illustrates ACH by room type and categorized based on whether or not AHU deficiencies were found. In areas where the AHU system was functioning properly, the median ACH was 12.9 (IQR = 14.8) in resident rooms and 10.4 (IQR = 14.6) in resident washrooms. When a single deficient component was found, the median ACH dropped to 4.6 (IQR = 11.1) in common areas, 1.8 (IQR = 9.3) in resident rooms and 7.1 (IQR = 7.2) in resident washrooms. In rooms where multiple deficiencies were present, further reductions were observed, with median ACH falling to 2.0 (IQR = 5.8) in common areas and 0 (IQR = 2.2 and IQR = 0, respectively) in both resident rooms and washrooms.

When the individual components were further examined, resident rooms without a ducted air source had a median ACH of 4.6 (IQR = 10.9), which further declined to 1.8 (IQR = 3.5) in rooms with closed dampers or inactive fans. In contrast, resident washrooms requiring only fan maintenance had a median ACH of 9.5 (IQR = 6.9).

Table 1 shows the association between ACH and AHU characteristics across different CLS room types. In resident rooms, the presence of closed outdoor air dampers and fans turned off were significantly associated with lower ACH (*P* = .047). Similarly, the absence of a ducted air source was significantly associated with reduced ACH in resident rooms (*P* < .05). In resident washrooms, closed outdoor air dampers and fans turned off were associated with a significant decrease in ACH (*P* < .05).

**Table 1.** Results of the multivariable linear regression analyses of specific air handling unit (AHU) characteristics on the number of air changes per hour (ACH) across congregate living rooms Table 1 long description.

Characteristics	Common area			Resident rooms			Resident washrooms		
	Change in ACH	*P*-value	95% CI	Change in ACH	*P*-value	95% CI	Change in ACH	*P*-value	95% CI
***Type of air supply***									
**Non-central system**	−3.64	.46	−14.39, 7.12	8.97	.11	−2.49, 20.42	−4.40	.14	−10.46, 1.65
**Hybrid system**	−3.39	.50	−14.30, 7.52	−2.28	.97	−14.27, 13.72	4.03	.29	−4.04, 12.11
**Dampers closed/fans off**	−3.05	.53	−13.81, 7.72	−9.54	.05	−18.91, −0.16	−6.98	.02	−12.66, −1.30
**Filters inadequate**	1.64	.73	−8.76, 12.03	NA	NA	NA	NA	NA	NA
**Suboptimal non-continuous supply airflow (fan set to AUTO instead of ON)**	0.92	.88	−12.39, 14.22	NA	NA	NA	NA	NA	NA
**Required maintenance**	−1.64	.74	−12.53, 9.24	NA	NA	NA	−4.58	0.11	−10.40, 1.24
**No ducted air source in resident rooms**	NA	NA	NA	−12.22	.04	−23.48, −0.97	NA	NA	NA

ACH, air changes per hour. CI, confidence interval.

## Discussion

In this cross-sectional study across 15 CLS, we identified a clear association between deficiencies with maintenance or design of AHU and lower ACH. Many such factors are considered modifiable and would have significant potential to improve the safety of these environments.

Some of the specific deficiencies of AHU have been recognized in prior studies. A recent systematic review of HVAC design features identified 23 studies that studied the effect of filtration on transmission of respiratory viruses, but these were limited to animal studies (n = 7), aerosolized virus studies (n = 7), and modeling studies (n = 9).^12^ A separate study assessed the most common types of HVAC maintenance-related deficiencies based on semi-structured interviews by HVAC experts.^13^ These authors found similar deficiencies, including dust accumulation on coils, clogged or improperly installed filters and air leakage in ducts. Finally, a recent study assessed a convenience sample of 10 long-term care facilities in California and found many similar problems. None of the facilities operated their HVAC systems continuously, only 40% had all outdoor air dampers open, just 20% used MERV-13 or higher-rated filters and only 20% performed directional airflow assessments.^14^

Our study categorized deficiencies by location and quantified their effects on ACH. In common area, the median ACH in common areas was 3.3, with only a quarter of the CLS meeting the minimum reference standards.^10,11^ Given the high traffic and occupancy of common areas, this represents an important opportunity for mitigation of viral respiratory transmission.^2^ In resident washrooms, exhaust fans are required to operate continuously yet we found that over a third of CLS had independent switches that could interrupt these fans.

We identified specific actions that Infection Prevention teams can take in collaboration with Plant Operations and HVAC experts to ensure ACH is optimized. These include first locating the AHU on-site to determine the configuration of outdoor air intake. From there, teams can ensure AHU components are operational including: outdoor air damper position allowing continued outdoor air volume while the unit is operational; filters changed according to the manufacturer’s recommendations; heating or cooling coils are clean and functioning properly; supply air fan is operational at all times; return air fan is operational at all times; return air pathways are unobstructed and functioning properly to maintain balanced airflow throughout the system; and exhaust systems are turned on and functioning as designed.

In contrast to maintenance and cleaning improvements, some deficiencies identified may not be feasible to address without construction or renovation. Lack of air ducting to resident rooms for example was observed in one third of CLS in our study. However, other readily actionable deficiencies remained over four-fold more frequent than lack of air ducts suggesting that there remains significant opportunities to optimize existing infrastructure as a first step.

Another barrier to undertaking improvements to HVAC systems is the lack of a clear framework similar to other types of environmental audits. Standards for CLS may also vary between countries.^10,11^ Our study findings can help facilitate this assessment, adapted in the form of a checklist that can be completed in collaboration with trained HVAC professionals and based on appropriate national standards (Supplemental material).

Our study has several important limitations. First, our findings may not be applicable to all types of CLS, though we included a diverse group of NH and ILF with variable sizes. Second, as a cross-sectional study, measurements were only taken once across all CLS and therefore any variation in air flow may not have been captured. Third, we cannot exclude the presence of other confounding factors affecting indoor ventilation, such as room occupancy at the time of assessment. Fourth, the ACH in common areas under fully functioning conditions could not be measured because all AHU serving these spaces had at least one deficient component at the time of assessment. Finally, only overall ACH was measured due to inability to accurately measure fresh ACH in some participating CLS. However, previous studies have found associations between overall ACH and viral respiratory transmission.^2,4^

In this cross-sectional study of HVAC across 15 CLS, we identified readily actionable targets for making improvements to ventilation. Future prospective studies of improvements to AHU on safety of NH and ILF residents are needed.

## Supporting information

10.1017/ice.2026.10458.sm001 Chan et al. supplementary material Chan et al. supplementary material

## Supplementary material

The supplementary material for this article can be found at https://doi.org/10.1017/ice.2026.10458.

## Figure 1 Long description

A diagram of an air handling unit showing the flow of air through various components. The diagram includes the following labeled parts: 1. Outdoor air, 2. Automated dampers, 3. Prefilter, 4. Heating coil/humidifier, 4. Cooling coil, 3. Final filter, 5. Supply air fan, 6. Indoor space, 7. Return air fan, 8. Return air, 9. Exhaust air. Outdoor air enters through automated dampers and passes through a prefilter. It then goes through a heating coil or humidifier and a cooling coil. The air is further filtered through a final filter before being supplied to the indoor space by a supply air fan. Air from the indoor space is returned through a return air fan and can either be exhausted outside or recirculated back into the system.

Navigate back to Figure 1.

## Table 1 Long description

The table presents the association between air changes per hour (ACH) and air handling unit (AHU) characteristics across different congregate living space (CLS) room types. It includes data for common areas, resident rooms, and resident washrooms. The table has 10 rows and 9 columns. The columns are labeled as Characteristics, Change in ACH, P-value, 95% CI for common areas, Change in ACH, P-value, 95% CI for resident rooms, and Change in ACH, P-value, 95% CI for resident washrooms. The rows list different types of air supply and their impact on ACH. Row 1: Non-central system, -3.64, .46, -14.39, 7.12, 8.97, .11, -2.49, 20.42, -4.40, .14, -10.46, 1.65. Row 2: Hybrid system, -3.39, .50, -14.30, 7.52, -2.28, .97, -14.27, 13.72, 4.03, .29, -4.04, 12.11. Row 3: Dampers closed/fans off, -3.05, .53, -13.81, 7.72, -9.54, .05, -18.91, -0.16, -6.98, .02, -12.66, -1.30. Row 4: Filters inadequate, 1.64, .73, -8.76, 12.03, NA, NA, NA, NA, NA, NA, NA, NA. Row 5: Suboptimal non-continuous supply airflow, 0.92, .88, -12.39, 14.22, NA, NA, NA, NA, NA, NA, NA, NA. Row 6: Required maintenance, -1.64, .74, -12.53, 9.24, NA, NA, NA, NA, -4.58, 0.11, -10.40, 1.24. Row 7: No ducted air source in resident rooms, NA, NA, NA, NA, -12.22, .04, -23.48, -0.97, NA, NA, NA, NA.

Navigate back to Table 1.
