# Supplementary material for: Assessment of air handling unit to improve ventilation in congregate living settings: a multicenter cross-sectional study
Source: Infect Control Hosp Epidemiol. 2026 May 11;47(7):692–6. doi: 10.1017/ice.2026.10458 (PMC13315534; doi:10.1017/ice.2026.10458)
Supplement: Chan et al. supplementary material [file S0899823X26104589sup001.docx]

**Assessment of Air Handling Unit in congregate living settings**

*This checklist is intended to serve as a best practice guide for assessment of Air Handling Unit (AHU) to support collaboration between Infection Prevention and Control (IPAC) and Heating, Ventilation, and Air Conditioning (HVAC) experts, and is to be completed annually before start of respiratory virus season.*

| **Date:** |  |
| --- | --- |
| **Facility name:** |  |
| **Location assessed:** |  |
| **Date of last HVAC inspection:** |  |
| **Assessor’s name(s):** |  |

1. ***Baseline HVAC measurements (if available)***

| ***Date of HVAC assessment*** | ***Type of Room***  ***(C = common area; R= resident room; W = resident washroom)*** | ***Outdoor ACH*** | ***Total***  ***ACH*** | ***Meets Standard****  ***(Yes or No)*** |
| --- | --- | --- | --- | --- |
|  |  |  |  |  |
|  |  |  |  |  |
|  |  |  |  |  |

** See Applicable Reference Standard below*

1. ***AHU assessment***

*Please check ✓ the box that applies to each component depicted in this simplified schematic.*


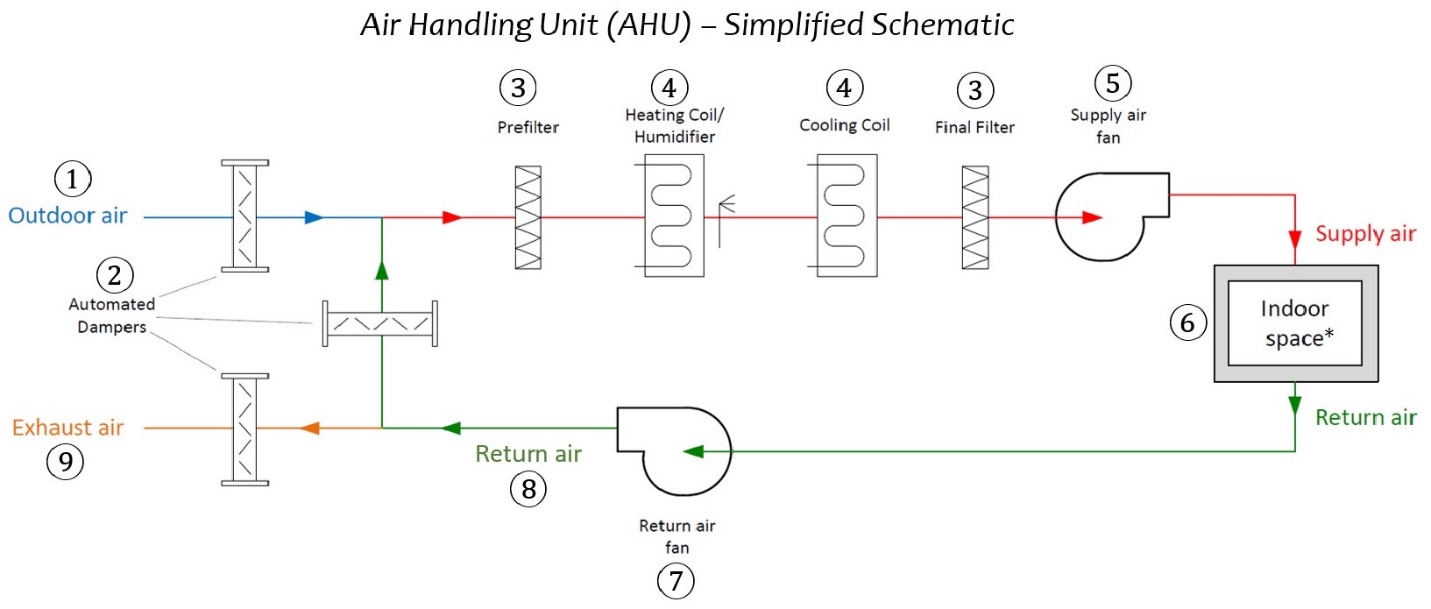


**Indoor space refers to resident/patient rooms (including washrooms) and common areas. Offices are excluded.*

|  |  |  | Compliant | |  |
| --- | --- | --- | --- | --- | --- |
| # | **Component*** | **Assessment Criteria** | **Yes** | **No** | **Comments** |
| ① | Outdoor Air | Location and configuration of the air handling unit support appropriate outdoor air intake (e.g. no construction activity nearby, presence of exhaust discharge vent, etc.). | ☐ | ☐ |  |
| ② | Automated Outside Air Dampers | Outside air dampers modulate the minimum outdoor air requirements (please refer to Appendix 1). | ☐ | ☐ |  |
|  | Automated Return Air Dampers | Return air dampers remain open and that return airflow adjusts in proportion to the supply and outside air to maintain proper air balance, operating conversely with the outside air dampers. | ☐ | ☐ |  |
|  | Automated Exhaust Air Dampers | Exhaust air dampers remain open and the airflow is balanced with the outside air dampers. | ☐ | ☐ |  |
| ③ | Prefilter | Filters have appropriate MERV rating (MERV 6-8) and are replaced according to the manufacturer’s recommendations including maintenance of frame housing as appropriate. | ☐ | ☐ |  |
|  | Final Filter | Filters have appropriate MERV rating (MERV 13 or higher) and are replaced according to the manufacturer’s recommendations.^1^ | ☐ | ☐ |  |
| ④ | Heating/Cooling Coils | Coils are clean and functioning properly. | ☐ | ☐ |  |
| ⑤ | Main Supply Air Fan | Supply air fan is turned ON and functioning at all times. | ☐ | ☐ |  |
| ⑥ | Indoor Space: Local Supply and Return Air Fan | Fan thermostat set to Fan ON (NOT in AUTO) during occupied hours. If digital controls are available, disable the AUTO mode; otherwise use a label to indicate this setting. | ☐ | ☐ |  |
| ⑦ | Main Return Air Fan | Return air fan is turned ON and functioning at all times. | ☐ | ☐ |  |
| ⑧ | Return Air | Return air pathways are unobstructed and functioning properly to maintain balanced airflow throughout the system. | ☐ | ☐ |  |
| ⑨ | Local Exhaust Air | Exhaust systems are functioning as designed and turned ON at all times (e.g. resident bathrooms). Fan should NOT be connected to light switches. Ideally, systems should use a central fan with ducted grills. | ☐ | ☐ |  |
|  | Main Exhaust Air | Exhaust systems are functioning at all times. | ☐ | ☐ |  |

**see definition below*

***AHU Definitions***

- ***Outdoor air:*** Air outside a building or taken from the external atmosphere and has not previously circulated through the AHU.
- ***Dampers:*** Elements inserted into an air-distribution system that permit modification of the air resistance, thereby changing the airflow rate or shutting off the airflow
- ***Pre-filter:*** The first stage of air filtration designed to remove large airborne particles.
- ***Heating coil/humidifier:*** Heating coils uses a heat transfer fluid, condensing refrigerant or direct electoral resistance elements to provide heating , while a humidifier adds moisture to the air.
- ***Cooling coil:*** An arrangement of pipes or tubes using refrigerant or secondary coolant to provide cooling or cooling with dehumidification.
- ***Final filter:*** A filter positioned in the last filtering position in an AHU to collect dust that has passed through.
- ***Supply air:*** Air delivered by mechanical or natural ventilation to a space, composed of any combination of outdoor air and recirculated return air.
- ***Return air:*** Air removed from a room to be recirculated or exhausted.
- ***Exhaust air:*** Air must be removed from a space due to contaminants.

**Ventilation reference standards**

| **Room Type** | **CSA standard^1^** | | **ASHREA standard^2^** | |
| --- | --- | --- | --- | --- |
|  | **Outdoor ACH** | **Total ACH** | **Outdoor ACH** | **Total ACH** |
| Common area | 2 | 6 | 4 | 4 |
| Resident room | 2 | 4 | 2 | 2 |
| Resident washroom | - | 9 | - | 10 |

^1^CSA Group. CSA Z317.2:19 Special requirements for heating, ventilation, and air-conditioning (HVAC) systems in health care facilities. Canada: Standards Council of Canada; 2019.

^2^American Society of Heating Refrigerating and Air-Conditioning Engineers, American National Standards Institute. ANSI/ASHRAE/ASHE Standard 170-2021. Ventilation of health care facilities, 2021. Available at <https://www.ashrae.org/file%20library/technical%20resources/standards%20and%20guidelines/standards%20addenda/170_2021_c_20210730.pdf>. Accessed February 27, 2026.
